# A comparative study of greener alternatives for nanocellulose production from sugarcane bagasse

**DOI:** 10.1186/s40643-021-00477-0

**Published:** 2021-12-19

**Authors:** Bhargavi Pula, Shradha Ramesh, Sirisha Pamidipati, Purnima Doddipatla

**Affiliations:** Department of Chemical Engineering, Birla Institute of Technology and Science Pilani, Hyderabad Campus, Jawahar Nagar, Shameerpet Mandal, Hyderabad, India

**Keywords:** ABTS, Glucose oxidase, Laccase enzyme, Nanocellulose, Optimization studies, Sugarcane bagasse

## Abstract

Use of enzyme for extraction of nanocellulose from sugarcane bagasse is greener alternative. Literature indicates that effectiveness of these enzymes can be improved by auxiliary enzymes or mediators. In the current study, extraction of nanocellulose using laccase with these moderators, auxiliary enzyme glucose oxidase and mediator molecule, ABTS [2,2′-azinobis(3-ethylbenzthiazoline-6-sulfonate)] individually was done. Cellulose and lignin content, FT-IR, TGA and DSC analysis, XRD, SEM and PSA were done. Enzyme moderators improved the performance of laccase in lignin degradation. Lignin and cellulose content, crystallinity were used as parameters to optimize the concentrations, which was found to be ABTS (at 1.4 mM) and glucose oxidase (at 0.15 mg ml^−1^). At the optimal concentration, nanocellulose was extracted. Properties of nanocellulose obtained from both routes were compared. Size analysis revealed 339 nm and 636 nm for nanocellulose obtained with glucose oxidase and ABTS, respectively. Defibrillation was better in the case of the former one as seen from SEM.

**Graphical Abstract:**

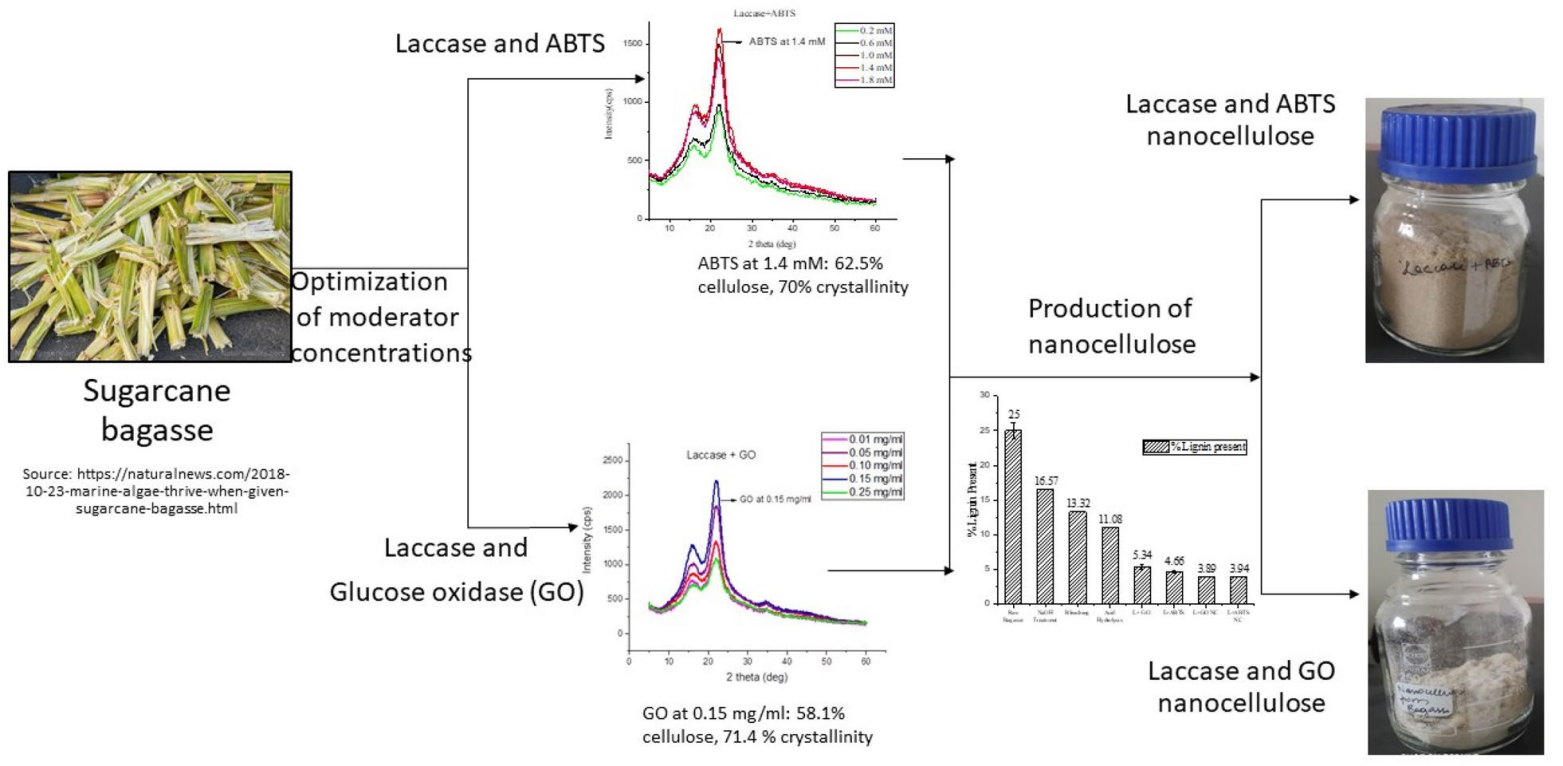

## Introduction

Lignocellulosic agricultural residues such as sugarcane bagasse are produced in large quantities every year (more than 1287 million tons) (Melati et al. 2017). Currently, the residue is mainly used as a fuel source to power sugar mills (Mandal and Chakrabarty 2011). A better alternative to using the biomass residues is for the production of nanocellulose, as cellulose constitutes 40–50% of its weight (Rocha et al. 2015; Yu et al. 2021). Nanocellulose has a wide range of applications in diverse industries such as biomedical and all engineering fields, owing to its physical and mechanical properties with rich surface chemistry and biocompatibility (Gupta and Shukla 2020; Khalid et al. 2021a). Nanocellulose has shown great interest due to exceptional qualities such as lower cost, lightweight, active surface functionalization, biodegradability, and eco-friendly nature (Khalid et al. 2021b). The application of nanocellulose in varied areas such as manufacturing, cosmetics, food, biomedical, and electronics sectors, has regarded it as a novel material with high value addition (Mandal and Chakrabarty 2011; Gupta and Shukla 2020; Yu et al. 2021). Use of sugarcane bagasse to produce nanocellulose will therefore serve as an apt example for a waste-to-wealth economy.

Lignocellulosic biomass primarily consists of cellulose, hemicellulose, and lignin. Lignin is a complex aromatic heteropolymer that consists of phenylpropanoid aryl-C3 units, linked together with ether and C–C bonds (Chandra et al. 2015). For nanocellulose production from any lignocellulosic biomass material, an important step is the removal of lignin, since most of the short and loose-chained hemicellulose fraction gets solubilized in the pre-treatment steps leaving behind modified lignin in the insoluble matrix (Pereira Ramos 2003; Gupta and Shukla 2020). This leads to decreased accessibility to cellulose fraction, subsequently hindering furthering processing of biomass fibres for nanocellulose production. Reports also suggest that lignin binds with the hydrolytic enzymes non-specifically and thus obstruct cellulose hydrolysis (Rahikainen et al. 2011). For extracting nanocellulose from lignocellulosic residues, conventional treatment methods include chemical, thermo-chemical or physicochemical methods. While chemical methods involve the use of strong chemicals, physical methods are not easily scalable (Mandal and Chakrabarty 2011; Pereira et al. 2011; Wulandari et al. 2016; Luzzi et al. 2017).

A better alternative to conventional methods in extracting nanocellulose from biomass is the use of enzymes (Tsegaye et al. 2018). Enzymes have been used for lignin removal in not just lignocellulosic biomass residues, but also the surface treatment of fibres used in polymer composites (Agrawala et al. 2019). Enzymatic hydrolysis is gaining much more research interest over other pre-treatment routes because of various advantages it offers, namely, higher specificity, a requirement of no chemicals, lower energy inputs, and an eco-friendly process (Siqueira et al. 2010). However, it is important to promote the activities of these enzymes by employing co-factors, mediators, etc., which in turn reduces the processing times. In the enzymatic treatment of biomass for lignin removal, laccases are considered to be most resourceful with broad specificity (Christopher et al. 2014; Flórez-Pardo et al. 2015). However, laccases can only oxidize phenolic lignin structures that have a lower redox potential (< 0.4–0.8 V) and substrates which are large and bulky pose permeability issues (Tadesse et al. 2008).

A promising alternative to increase the laccase activity and broaden its substrate specificity is using the enzyme along with mediators, which are essentially low-molecular-weight molecules capable of intervening in the laccase-mediated redox reactions (Christopher et al. 2014; Moilanen et al. 2014). Such mediator molecules are capable of diffusing through smaller pores of the lignin matrix and thus reduce the diffusional resistance between the enzyme and substrate molecules. In presence of mediator molecules such as ABTS [2,2′-azinobis(3-ethylbenzthiazoline-6-sulfonate)], laccases were found to oxidize even non-phenolic lignin model compounds (Moilanen et al. 2014; Rajak and Banerjee 2015).

Apart from studies on laccase mediators, researchers have studied the laccase mechanism and concluded that laccase secretes quinonoid intermediates as a result of its action on phenolic substrates of lignin (Curl et al. 2001; Witayakran and Ragauskas 2009). These quinonoid radicals block the active site of the laccase enzyme or repolymerize to form lignin polymer, thus decreasing its efficiency of lignin degradation (Rodríguez-Escribano et al. 2017). Glucose oxidase (GO) helps to transform the quinones into simpler phenolic compounds and also prevents repolymerization of lignin from lignin-degraded intermediates (Szklarz and Leonowicz 1986; Leonowicz et al. 2001). Hence, enzymatic pre-treatment methods may offer greener alternatives that are more economical with lesser processing times and higher yields (Flórez-Pardo et al. 2015; Michelin et al. 2020).

In the current study, two strategies of increasing the laccase activity, namely: (a) by using mediators that reduce diffusional resistance and broadens substrate specificity; and (b) using oxidase enzymes that work concomitantly with laccase, blocking quinonoid intermediates and preventing repolymerization of lignin have been investigated. For the study, laccase has been used to treat sugarcane bagasse using two kinds of supplements/moderators: (a) ABTS mediator, and (b) glucose oxidase enzyme. In the initial experiments, the concentrations of the laccase moderators, namely glucose oxidase and ABTS were optimized. For these optimization studies, lignin and cellulose content before and after treatment, XRD analysis to study structural differences were performed and analysed. Later, at the optimal concentrations of moderators, nanocellulose was extracted from mildly treated sugarcane bagasse and further characterized to compare the efficiency of the two laccase moderators.

## Material and methods

### Materials

Raw sugarcane bagasse was obtained from a local vendor and was thoroughly washed in water multiple times and dried at 80–90 °C. It was further chopped into smaller pieces with an average size of 2–3 cm. The material was stored in a closed container till further use (Ramesh et al. 2021). The chemical reagents, sodium hydroxide, hydrogen peroxide, and oxalic acid were all analytical grade (AR grade, 95% purity) and obtained from SD Fine Chemicals, Mumbai.

For the enzymatic treatment, pure laccase enzyme (source: *Trametes versicolor*, Product No: 51639-1G, Activity: 12.9 U mg^−1^) and pure glucose oxidase enzyme (source*: Aspergillus Niger*, Product No: G7141-10KU, Activity: 100–250 U mg ^−1^) were obtained from Sigma-Aldrich. Laccase mediator, 2,2-azino-bis (3-ethylbenzothiazoline-6-sulfonate) di-ammonium salt (ABTS) (Product No: A1888-1G) was also obtained from Sigma-Aldrich.

### Methods

#### Chemical pre-treatment

Sugarcane bagasse was treated with sodium hydroxide 2% (w/v) for 6 h at 25 °C and oven-dried for 6 h (De Campos et al. 2013). The dried bagasse was then bleached with hydrogen peroxide solution 30%(w/v) maintained at pH 2.3 for 6 h at 25 °C and oven-dried for 6 h (Azzam 1989) followed by mild acid hydrolysis with oxalic acid at 1% (w/v) concentration (Abraham et al. 2013). Finally, the bagasse was thoroughly washed with water several times to remove residues of chemicals before further treatment.

#### Enzyme treatment

##### For optimization

For enzymatic treatment, 10 g of raw bagasse was treated with various combinations of enzymes: (a) laccase and glucose oxidase, and (b) laccase and ABTS for 48 h during which the pH was maintained at 5 with 10 mM citrate buffer solution. The concentration of laccase was optimized in a separate study and was found to be 0.015 mg ml^−1^. Similarly, the concentration of glucose oxidase was varied between the range of 0.025–0.25 mg ml^−1^, and the optimum value was recorded at 0.15 mg ml^−1^. The concentration of ABTS was varied between the 0.2 and 2 mM range and the optimized value was determined at 1.4 mM.

##### For nanocellulose production

For enzymatic treatment, mild acid hydrolysed bagasse was treated with various optimal combinations of enzymes: (a) laccase and glucose oxidase (at 0.15 mg ml^−1^); and (b) laccase and ABTS (at 1.4 mM) for 48 h during which the pH was maintained at 5 with 10 mM citrate buffer solution.

#### Extraction of nanocellulose

**Fig. 1:**
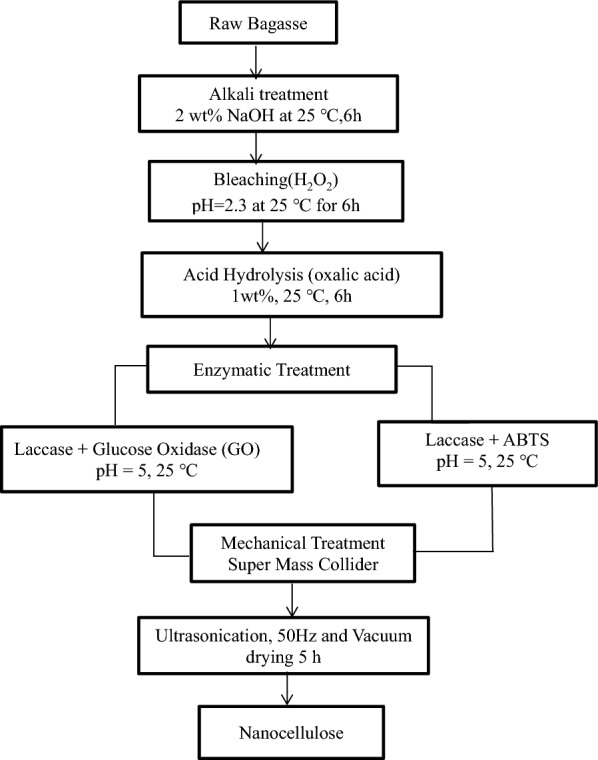
Schematic representation of the nanocellulose synthesis

Nanocellulose was extracted from pretreated sugarcane bagasse as detailed in our previous paper (Ramesh et al. 2021). Figure 1 summarizes the steps that were followed to produce nanocellulose from the raw bagasse. For mechanical treatment, the bagasse was soaked for 24 h in water and later treated in Super Mass Collider (Masuko Sangyo MKCA6-2 J) to yield nanocellulose. The rotational speed was slowly increased from 500 to 700 rpm to prevent friction between the grinding stones and dissipate heat. The mechanical treatment is done to reduce the size of the fibre to the nano-range. The agglomerated suspension was sonicated (EN-50-US) for 3–4 h at 50 Hz and kept at 50 °C to stabilize the particle and generate the nanocellulose. The suspension was later vacuum dried for 5 h to remove moisture content (Kumar et al. 2014; Ramesh et al. 2021).

#### Lignin estimation

0.25 g of sugarcane bagasse was taken in a conical flask and 5 ml of 72% sulphuric acid was added and the sample was incubated for 2 h at 20 °C. Subsequently, 192.5 ml of distilled water is added and autoclaved at 121 °C for 1 h. The sample was filtered, and the residual insoluble lignin was thoroughly washed and was oven-dried till constant weight. The filtrate, soluble lignin was measured in UV spectrometer to obtain the absorbance value (Viera et al. 2007; Ramesh et al. 2021):

1a $${\text{Insoluble weight content(}}\% ) = \frac{{W_{{{\text{lignin}}}} }}{{W_{{{\text{fibre}}}} }} \times 100,$$

1b $${\text{Soluble weight content (}}\% ) = \frac{{{\text{Conc}} \times V_{{{\text{final}}}} }}{{W_{{{\text{fibre}}}} \times 1000}} \times 100,$$

where Conc = concentration of soluble lignin in the filtrate (g/l), *V*_final_ = total volume of the filtrate (ml).

$$C = \frac{A}{ \in \times L},$$

where *A* = absorbance value measured from the UV spectrometer, $\in$  = absorption coefficient (M^−1^ cm^−1^) − 105 for sugarcane bagasse, *C* = concentration of soluble lignin (M), *L* = path length (cm), insoluble lignin percentage + soluble lignin percentage = total lignin percentage.

#### Cellulose estimation

0.5 g of sugarcane bagasse was taken in a round-bottom flask. 15 ml acetic acid and 1.5 ml concentrated nitric acid was added to the sample and refluxed in a Soxhlet setup. The sample was then washed with ethanol and filtered using a cotton cloth followed by oven drying and its weight was noted (Material A). Material A was incinerated at 525 °C in the muffle furnace till constant weight was attained (Material B) (Yarbrough et al. 2017; Ramesh et al. 2021):

2 $$\% \,{\text{Cellulose}} = \frac{{{\text{Material}}\,{\text{A}} - {\text{Material}}\,{\text{B}}}}{{\text{Initial weight}}} \times 100.$$

#### Fourier-transform infrared (FT-IR) spectroscopy

The FT-IR spectra of the solid samples before and after every treatment were acquired on FT/IR 4200 spectrophotometer of JASCO Make. Sample pellets were prepared using 1 mg of sample and 100 mg of oven-dried potassium bromide. For each spectrum, 64 scans in the range of 400–4000 cm^−1^ with a resolution of 2 cm^−1^ were conducted in the transmittance mode and FT-IR spectra for the samples lie between ranges of 500–4500 cm^−1^ (Mandal and Chakrabarty 2011; Pamidipati and Ahmed 2019).

#### X-ray diffraction (XRD)

XRD was used to find the crystallinity of bagasse after every treatment process. The samples were analysed by Rigaku Ultima IV X-ray diffractometer, to find the crystallinity index at an angle range of 2*θ* = 5–60^o^ with a monochromatic CuKα radiation source at 30 kV and 20 mA with a step size of 0.02 and rate of increase at 1°/min (Ghazy et al. 2016).

#### Differential scanning calorimeter (DSC)

DSC is used to study the thermal behaviour of the cellulose sample at each treatment process. The sample was heated from 30 to 400 °C at a heat rate of 10 °C min^−1^ under a nitrogen atmosphere with a flow rate of 100 mL min^−1^ to avoid thermos-oxidative degradation (Mandal and Chakrabarty 2011).

#### Thermo-gravimetric analysis (TGA)

TGA is used to study the thermal stability of the cellulose sample. Weighted amount of 2.60 mg of sample was heated from 30 to 400 °C with the heating rate of 10 °C min^−1^ under nitrogen atmosphere with the flow rate of 100 mL min^−1^ to avoid thermo-oxidative degradation. The thermogram was used to determine the onset melting temperature of the sample (Mandal and Chakrabarty 2011).

#### Scanning electron microscopy (SEM)

SEM was used to study the morphology of fibre. The sample images were captured using Hitachi S3700 VP-SEM. The samples were silver-coated using the sputtering technique (Oliveira et al. 2016) and the images were captured for analysis.

#### Particle size analyzer (PSA)

Particle size was measured using ZEN MELVERN. The suspended fibres were placed in a cuvette and placed inside the analyzer which gave the average size of the particles. The particle range lies in between 300 and 500 nm under the following conditions: particle refractive index 1.33, viscosity 0.8872 cP, and temperature of 25 °C. Three measuring cycles of 5 min each were recorded and the average was taken using the software (DTS, Ver. 3.00 from Malvern (Mandal and Chakrabarty 2011).

## Results and discussion

### Optimization of the moderator concentration for optimal laccase activity

#### Klason’s lignin test

**Fig. 2:**
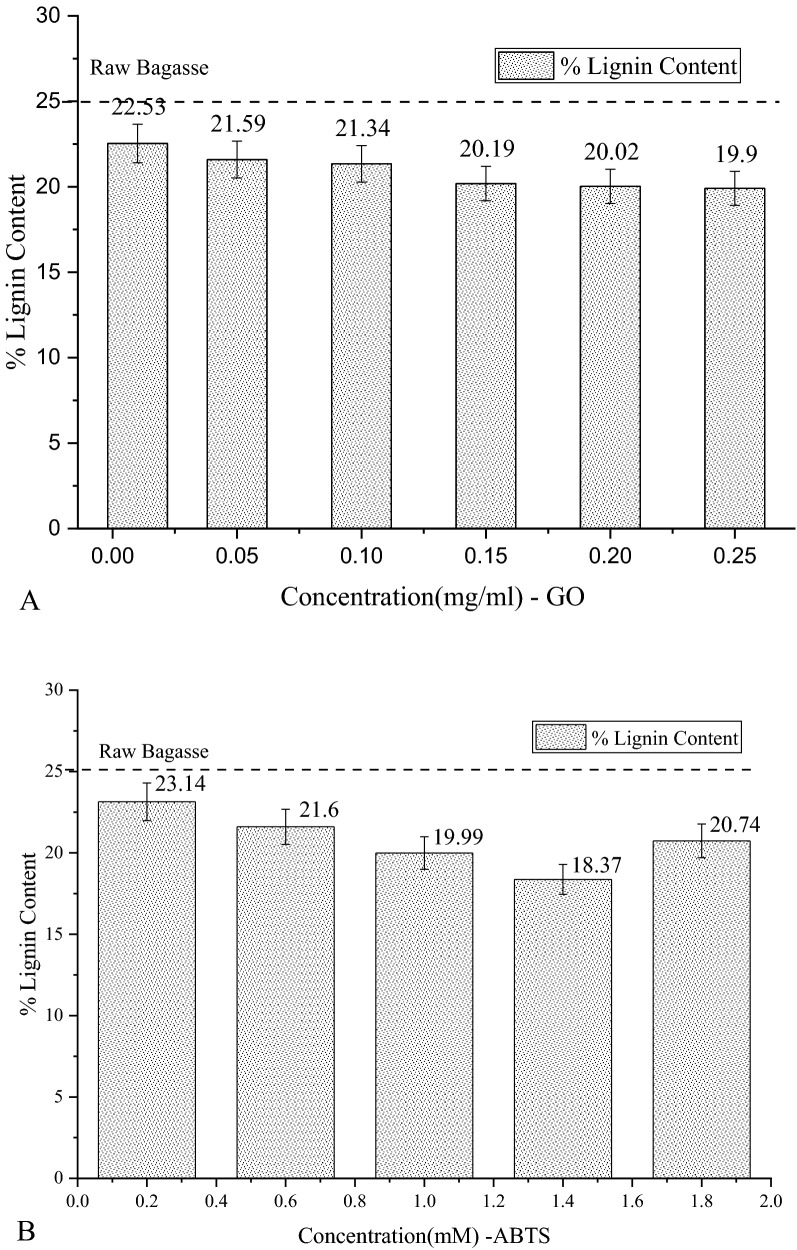
**A** Percentage of lignin present in sugarcane bagasse at different concentrations of glucose oxidase (mg ml^−1^). **B** Percentage of lignin present in sugarcane bagasse at different concentrations of ABTS (mM)

Klason’s lignin test was performed to optimize the various concentrations of glucose oxidase (GO) and ABTS when combined with the laccase (L) enzyme. The concentration of laccase was previously optimized in an earlier study as reported above, and the optimum concentration of laccase used for this entire study is 0.015 mg ml^−1^. Different concentrations of glucose oxidase and ABTS were taken and used to treat raw bagasse directly. From Fig. 2A, we observe that for varying concentrations of the glucose oxidase, the maximum reduction in the lignin content (from 25 for raw bagasse to 19.9%) was obtained at the GO concentration of 0.25 mg ml^−1^. From Fig. 2B, we see that for the different concentrations of the ABTS mediator, the maximum removal of lignin percentage was found at 1.4 mM. Beyond this concentration, the lignin content was found to increase again. Certain reports suggest that ABTS at higher concentrations can have an inhibitory effect on the laccase enzyme (Tavares et al. 2008), and therefore we may have seen a decreased lignin degradation at higher concentrations. Before choosing the optimal values of GO and ABTS, cellulose analysis of bagasse before and after treatment with the enzyme solution was also performed.

#### Cellulose test

**Fig. 3:**
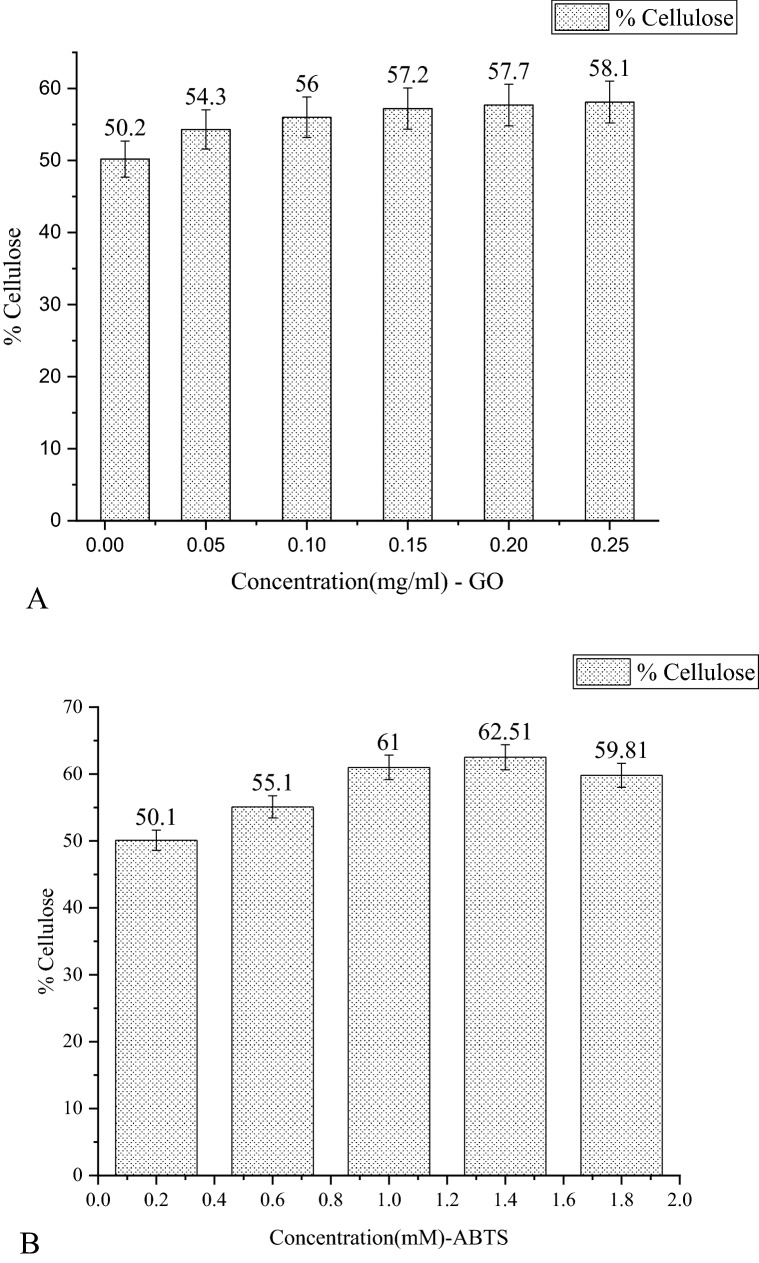
**A** Cellulose percentage in sugarcane bagasse for various concentrations of glucose oxidase. **B** Cellulose percentage in sugarcane bagasse for various concentrations of ABTS

Cellulose test was performed to optimize the various concentrations of glucose oxidase (GO) and ABTS when combined with the laccase (L) enzyme. The optimum concentration of laccase used for this entire study is 0.015 mg ml^−1^. Different concentrations of glucose oxidase and ABTS were used in combination with laccase to treat the raw bagasse for lignin removal. Figure 3A shows the cellulose content in raw bagasse (50.2%) and after treatment with laccase and GO, while Fig. 3B illustrates the variation in cellulose content for various concentrations of ABTS in presence of laccase. We can observe from both the figures that cellulose content increased after treatment with the enzyme solutions. While the raw bagasse has 50.2% cellulose content, it gradually increased as the GO concentration was increased, and the highest cellulose value was 58.1% at the concentration of 0.25 mg ml^−1^ GO and laccase (Fig. 3A). When raw bagasse was treated with varying concentrations of ABTS in presence of laccase (Fig. 3B), the cellulose content increased with an increase in ABTS concentration from 0.2 to 1.4 mM, and then decreasing trend was noted like the trend seen in lignin content (Fig. 3B). At 1.4 mM ABTS concentration the highest value of cellulose content, 62.51% was recorded. From the studies done on lignin and cellulose content, the optimal value for laccase and GO was found to be 0.25 mg ml^−1^ while for laccase and ABTS, 1.4 mM was the optimum concentration.

#### X-ray diffraction (XRD)

**Fig. 4:**
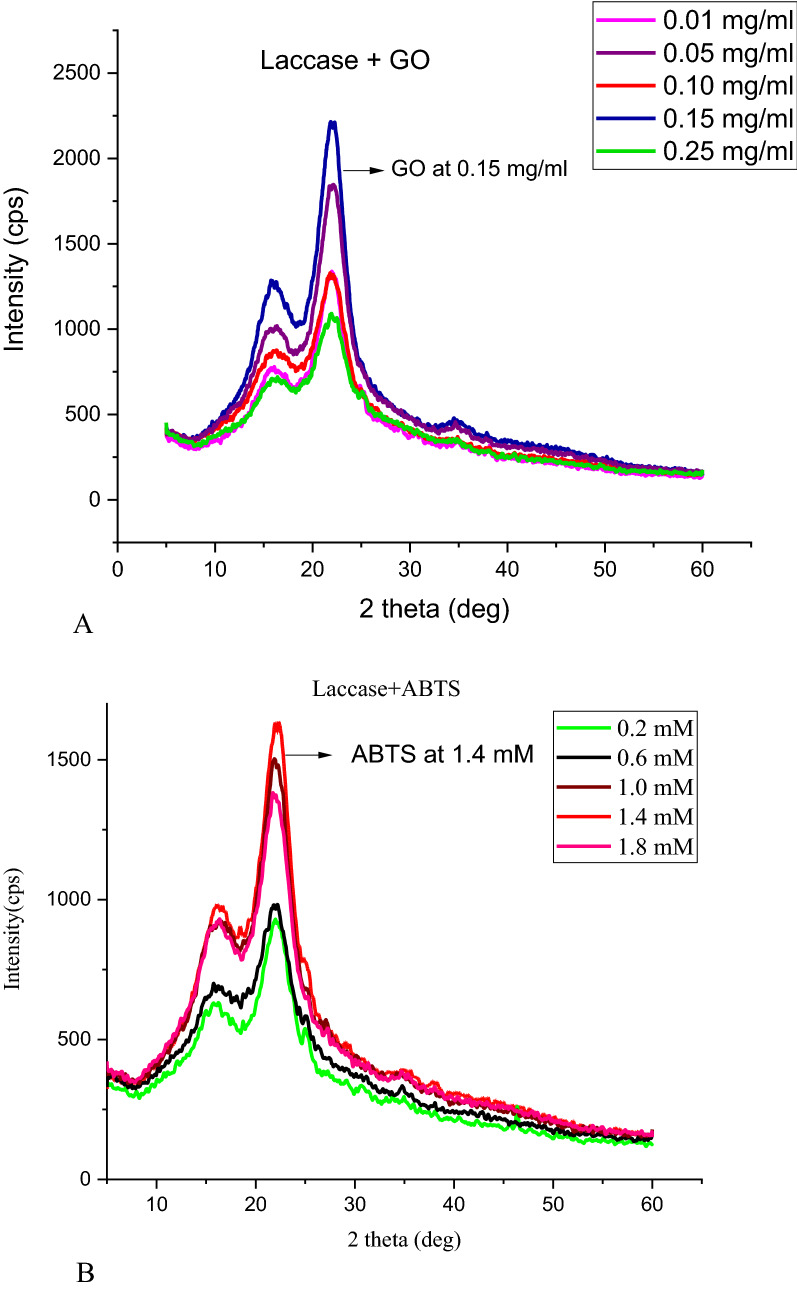
**A** X-ray diffractogram for different concentrations of GO with laccase. **B** X-ray diffractogram for different concentrations of ABTS with laccase

Apart from the percentage cellulose content, another important factor that needs to be considered for nanocellulose production is the degree of crystallinity in cellulose which will impact the final nanocellulose product. For this reason, XRD analysis was also performed on various combinations of GO and ABTS with laccase before finalizing the optimal concentrations. Figure 4A and B shows the XRD graphs for various combinations of GO with laccase and ABTS with laccase, respectively. From the figures, we can see that the highest intensity was noted at 0.15 mg ml^−1^ for glucose oxidase (Fig. 4A), while for ABTS, the crystallinity was highest at 1.4 mM concentration (Fig. 4B). The crystallinity indices for various concentrations were also calculated using Eq. (1), where intensity *I*_200_ indicates the crystalline compounds and *I*_am_ represents the amorphous compounds (Table 1). From Table 1, for the combination of laccase and GO, the maximum crystallinity of 71.4% was observed at a concentration of 0.15 mg ml^−1^. However, at previously considered optimal value of 0.25 mg ml^−1^ GO the crystallinity percent was found to be 60%. On the other hand, for ABTS, 1.4 mM concentration showed the highest crystallinity index at 70% and was consistent with the previous optimal values for lignin and cellulose content. Therefore, after analysing Klason’s lignin content, cellulose content, and XRD analysis, we optimized the glucose oxidase enzyme concentration to be 0.15 mg ml^−1^ and ABTS concentration as 1.4 mM. These concentrations were used with laccase (at 0.015 mg ml^−1^) for further studies on nanocellulose production and its characterization:

3 $${\text{Crystallinity Index }}({\text{C}}.{\text{I}}) - \left( {{{I_{200} - I_{{{\text{am}}}} } \mathord{\left/ {\vphantom {{I_{200} - I_{{{\text{am}}}} } {I_{200} }}} \right. \kern-\nulldelimiterspace} {I_{200} }}} \right) \times 100.$$

**Table 1:** XRD crystallinity indices for different concentrations of GO and ABTS when combined with laccase

S. no.	Conc. of GO (mg ml^−1^)	Crystallinity index (%)	Conc. of ABTS (mM)	Crystallinity index (%)
1	0.01	63.8	0.2	60
2	0.05	68.57	0.6	55.8
3	0.1	60	1.0	61.5
4	0.15	71.4	1.4	70
5	0.25	60	1.8	57

### Nanocellulose production and characterization

#### Klason’s lignin test and cellulose test

**Fig. 5:**
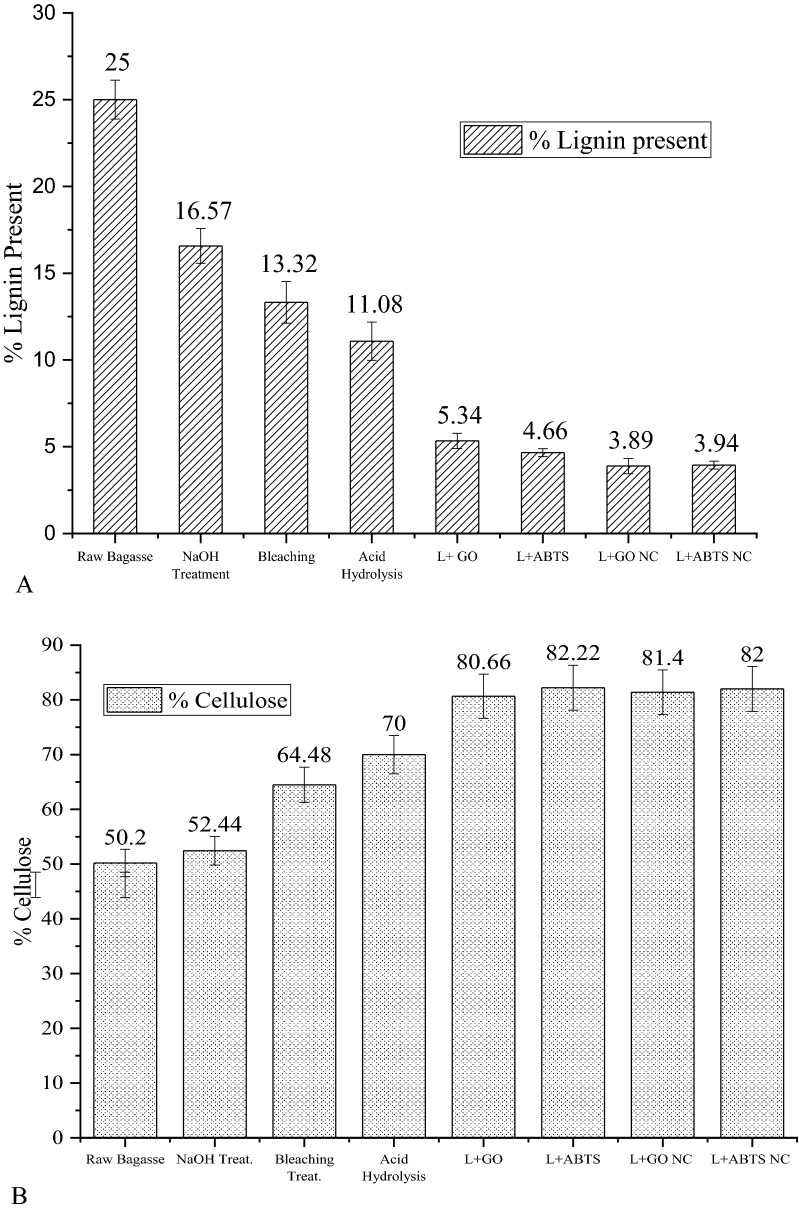
**A** Lignin percentage present after each treatment process. **B** Cellulose percentage after each treatment process

Nanocellulose was extracted from sugarcane bagasse as detailed in the materials and methods section. In the enzymatic treatment, the concentration of laccase used was 0.015 mg ml^−1^ and the optimized concentration of GO and ABTS, i.e., 0.15 mg ml^−1^ and 1.4 mM, respectively, were used. Klason’s lignin test and Cellulose tests were performed after each treatment process and reported in Fig. 5A and B. A significant removal in lignin was noted at each treatment step (Fig. 5A), and the decrease was from 25% originally present in bagasse to ~ 5% after enzyme treatment. The maximum lignin removal was observed at the enzyme treatment step, with laccase + ABTS at 52% and laccase + GO with 58% lignin removal. The lignin content in the final nanocellulose product obtained with laccase + GO and laccase + ABTS treatments are 3.89 and 3.94%, respectively. In literature, lignin removal by laccases in the presence of ABTS has been reported to be between 20 and 35% for 5–10 days for untreated biomass (Karp et al. 2015). In another study, up to 27% lignin removal was found in sugarcane bagasse with the laccase-mediator pre-treatment step (Rencoret et al. 2017). In our case, in the alkali and acid-pretreated sugarcane more than 50% of lignin removal was found with both laccase + ABTS and laccase + GO treatments.

The cellulose content was found to increase after every treatment process as shown in Fig. 5B. The cellulose content increased from 44.32 to 80.66% when treated with laccase + GO enzyme and has increased to 82.22% when treated with laccase + ABTS. However, when cellulose content was measured in the final nanocellulose product obtained after mechanical treatment, the cellulose content was almost similar for both the enzyme treatments. In the case of laccase + ABTS, a slight decrease in the cellulose content of nanocellulose was noted. We speculate that some of the crystalline bonds might have ruptured due to which the amount of cellulose content reduced after subjecting to mechanical treatment.

#### XRD analysis

**Fig. 6:**
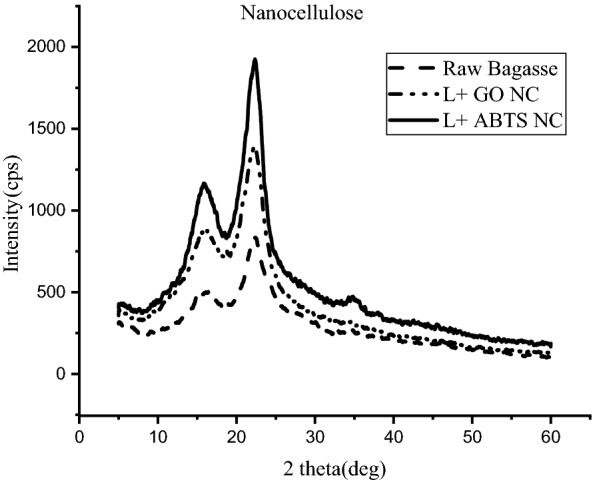
X-ray diffractograms of raw sugarcane bagasse and nanocellulose

XRD analysis was performed for nanocellulose obtained after the two enzyme treatments for calculating and comparing the crystallinity indices of cellulose. Figure 6 shows diffractogram obtained from raw bagasse and nanocellulose extracted from laccase + GO and laccase + ABTS. The two major diffraction peaks at the following 2$\theta$ angles, 15.2° and 22.5° represent the presence of cellulose-I structure. A clear increase in the intensities is seen for nanocellulose as compared to raw bagasse. When the intensities for the two enzyme treatments are compared, laccase + ABTS shows a higher intensity compared to laccase + GO. Also, the crystallinity indexes were calculated from Eq. (4) using the intensity at the 200 peak (I_200_, 2$\theta$ = 22.5°) and the minimum between the 200 and 110 peaks (I_am_ 2$\theta$ = 18°) (Mandal and Chakrabarty 2011). The crystalline index for raw bagasse was found to be 43.35% which increased to 59.42% in the case of laccase + GO nanocellulose and 61.71% laccase + ABTS nanocellulose (Table 2):

4 $${\text{Crystallinity index }}({\text{C}}.{\text{I}}) - \left( {{{I_{200} - I_{{{\text{am}}}} } \mathord{\left/ {\vphantom {{I_{200} - I_{{{\text{am}}}} } {I_{200} }}} \right. \kern-\nulldelimiterspace} {I_{200} }}} \right) \times 100.$$

**Table 2:** Crystallinity percentage of cellulose from XRD analysis and S/G ratio of lignin from FT-IR analysis

Sample	Crystallinity (%)	S/G ratio
Raw bagasse	43.35	0.892
Nanocellulose from laccase + GO treatment	59.42	2.01
Nanocellulose from laccase + ABTS treatment	61.71	1.56

#### Fourier-transform infrared (FT-IR) for cellulose crystallinity

**Fig. 7:**
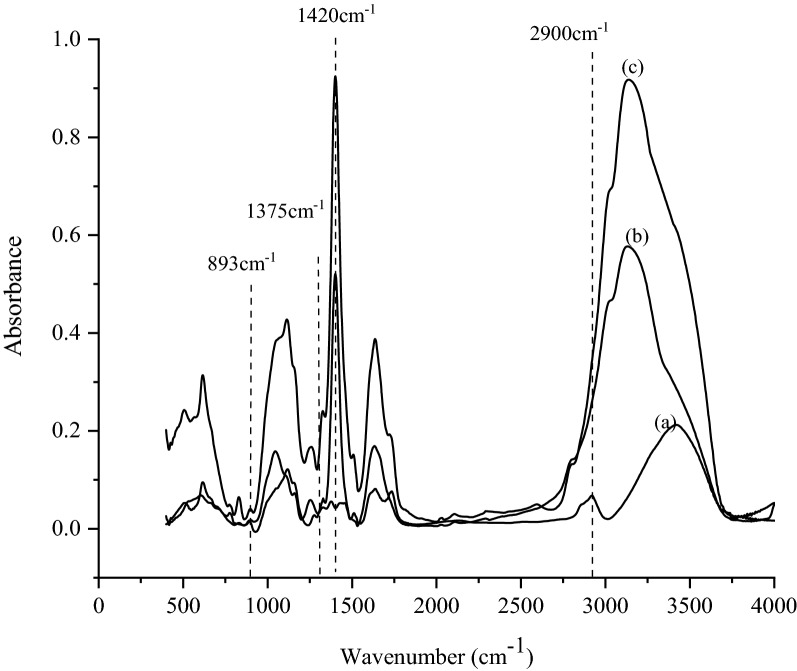
FT-IR spectra of **A** raw bagasse **B** nanocellulose from laccase + GO and **C** nanocellulose from laccase + ABTS

FT-IR analysis was also performed on raw bagasse, nanocellulose obtained from laccase + GO and laccase + ABTS (Fig. 7). The spectral bands seen at 1420 cm^−1^ and 893 cm^−1^, show the presence of cellulose-I content and describes the nature of crystal structures (Fan et al. 2012). Spectral analysis can be applied to the samples containing cellulose-I, II, and III or for the mixture of all the components (Kumar et al. 2014). In the spectra, the band between 1420 and 1430 cm^−1^ is known as ‘crystalline band’, while the band appearing between 893 and 898 cm^−1^ represents ‘amorphous band’ (Karimi and Taherzadeh 2016). To compare the degree of crystallinity in cellulose in different samples, the crystallinity indices, Lateral Order Index (LOI) and Total Crystallinity Index (TCI) were calculated by noting the absorbance values and taking ratios such as (Pamidipati and Ahmed 2019). Spectral ratio, 1420/893 cm^−1^ indicates LOI which is sensitive to the amount of crystalline versus amorphous regions in the cellulose, and therefore a lower value indicates more amorphous regions in the cellulose structure. The ratio 1375/2900 cm^−1^ represents TCI, which compares cellulose I, II, III, and amorphous cellulose (Fan et al. 2012). Table 3 shows the values of crystallinity indices, and these values were higher in nanocellulose as compared to raw bagasse. Also, nanocellulose obtained from laccase + GO (17.12) showed a significantly higher LOI in comparison to laccase + ABTS (13.75). However, when TCI values were compared the values were in the reverse trend with laccase ABTS (2.01) showing a higher value as compared to laccase + GO (1.19) which agrees with the trend seen in XRD analysis. Nanocellulose obtained from laccase + ABTS had slightly higher cellulose crystallinity as compared to laccase + GO.

**Table 3:** Lateral order index (LOI), total crystallinity index (TCI), from FT-IR analysis

Sample	Lateral order index (LOI) (1430/897 cm^−1^)	Total crystallinity index (TCI) (1375/2900 cm^−1^)
Raw bagasse	3.033	0.9268
Nanocellulose from laccase + GO	17.12	1.1875
Nanocellulose from laccase + ABTS	13.75	2.006

#### Fourier-transform infrared (FT-IR) for lignin degradation

FT-IR studies were also conducted to examine the changes in the chemical composition of the lignin in bagasse before and after enzyme treatment. Lignin consists of three monomers, namely p-hydroxyphenyl (H), guaiacyl (G) and syringyl (S) units (Fan et al. 2012). The degree of condensation and hence the ease of degradation of lignin can be studied by knowing the amounts of guaiacyl (G) and syringyl (S) content in lignin substructures. In this regard, S/G ratio which varies widely among various plant fibres is studied. In the paper and pulp industry, S/G ratio is widely used to study the degree of degradation of the lignin content and an increase in the ratio indicates increased ease of degradation of lignin as the more condensed guaiacyl linkages are broken down (Fan et al. 2012). In FT-IR spectrums, the S ring and the G ring stretching takes place at 1335 cm^−1^ and 1275 cm^−1^, respectively, and the ratio, S/G can be calculated from noting the absorbance values at these wavelengths (Fig. 7) (Pamidipati and Ahmed 2017). From Table 2 (S/G ratio), the ratio was found to increase after the enzymatic treatment process and laccase + GO reported a higher value as compared to laccase + ABTS. An increase in S/G ratio indicates a reduction in the guaiacyl content and therefore we speculate that laccase in combination with GO can degrade the more condensed guaiacyl linkages as compared to laccase with ABTS. An increase in S/G values with a reduction in lignin has also been reported in literature (Chanoca et al. 2019; Machado et al. 2020).

#### Scanning electron microscopy (SEM)

**Fig. 8:**
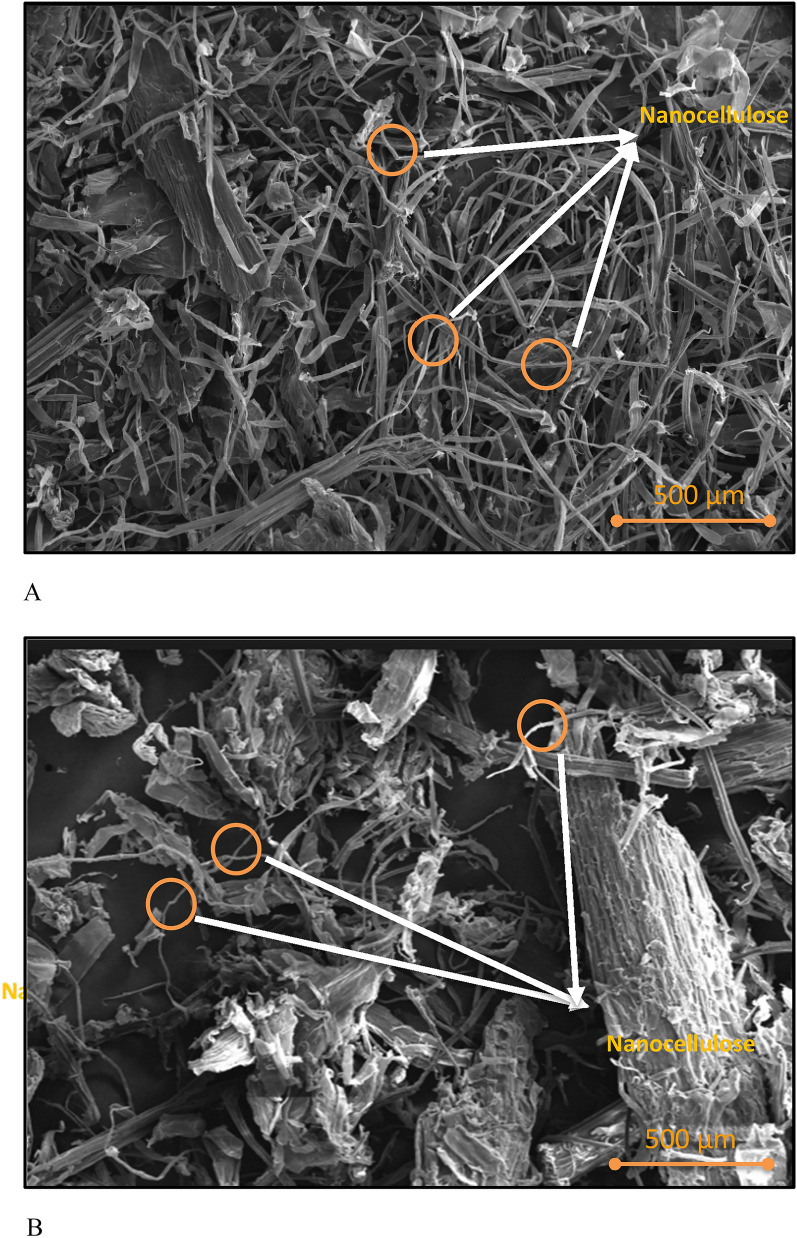
**A** SEM images for the nanocellulose–laccase + GO. **B** SEM images for the nanocellulose–laccase + ABTS

The scanning electron micrographs of nanocellulose obtained after: (a) laccase + GO, and (b) laccase + ABTS treatments are shown in Fig. 8A and B. Although the separation of fibres can be confirmed for both GO and ABTS, the separation can be considered to be distinctly good when GO has been used (Fig. 8A). The presence of certain agglomerated masses was noted in laccase + ABTS (Fig. 8B) nanocellulose. The clean, smooth surfaces seen in the case of laccase + GO indicate better defibrillation, probably due to the synergistic effect of the two enzymes, i.e., GO and laccase. The combination of laccase and GO enzymes effectively produced even and smooth nanocellulose fibres due to the removal of other components such as wax, pectin, lignin, and hemicellulose (Kumar et al. 2012). Also, the aspect ratio (L/D) of these fibres was calculated and found to be 35.84 for laccase + GO and 26.87 for laccase + ABTS. Literature reports the typical aspect ratios to be between 10 and 40 nm and the dimensions of nanocellulose obtained by both the treatments were well within this range (Kumar et al. 2012; Rahimi Kord Sofla et al. 2016).

#### Particle size analysis

**Fig. 9:**
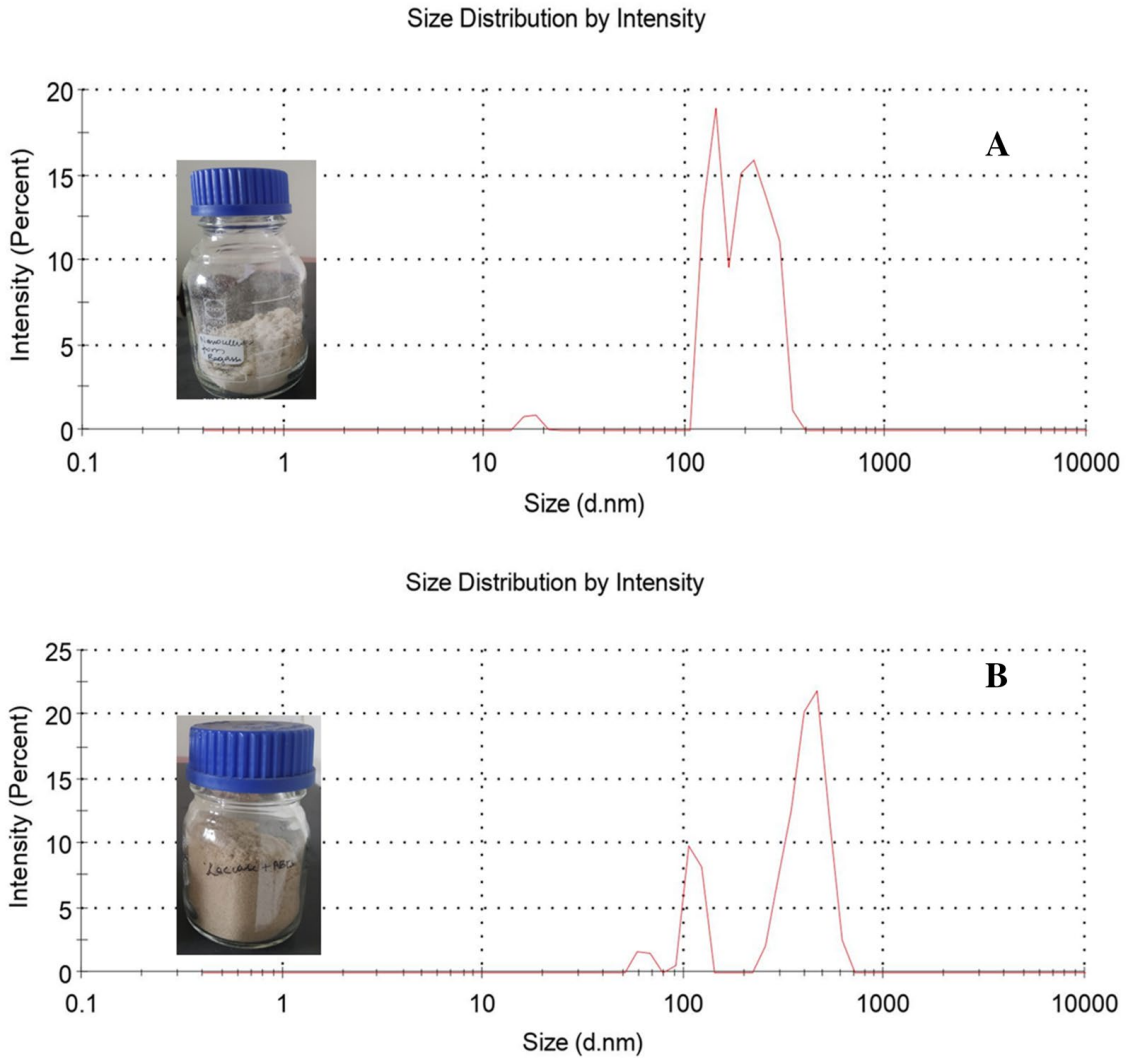
Particle size analysis of nanocellulose for **A** laccase + GO and **B** laccase + ABTS. The inset shows extracted nanocellulose in containers

The particle size distribution was measured by using the Particle size analyzer (PSA) and the size distribution is shown in Fig. 9A and B. The particle sizes were measured after the mechanical treatment (super mass collider) step. From Table 4, around 90% particle size distribution (by volume) was found within 285 nm for laccase + GO nanocellulose and 524 nm for laccase + ABTS. Moreover, the size distribution was found to be narrower for laccase + GO (Fig. 9A) as compared to laccase + ABTS (Fig. 9B). Thus, although nano-range dimensions can be observed for both GO and ABTS nanocellulose, it is evident that a narrower size distribution and better size reduction were found for laccase + GO as compared to that of laccase + ABTS (Fig. 9A and B). Also, as seen in Fig. 9 inset images, nanocellulose product obtained from laccase + GO was brighter in colour and the treatment with laccase + ABTS resulted in a light brown coloured nanocellulose.

**Table 4:** Particle size distribution for nanocellulose

Nanocellulose	Diameter (10) (≤ 10%)	Diameter (50) (≤ 50%)	Diameter (90) (≤ 90%)	Diameter (99) (≤ 99%)
Laccase + GO	(≤ ) 125 nm	(≤ ) 189 nm	(≤ ) 285 nm	(≤ ) 339 nm
Laccase + ABTS	(≤ ) 108 nm	(≤ ) 383 nm	(≤ ) 524 nm	(≤ ) 636 nm

#### Differential scanning calorimeter (DSC)

**Fig. 10:**
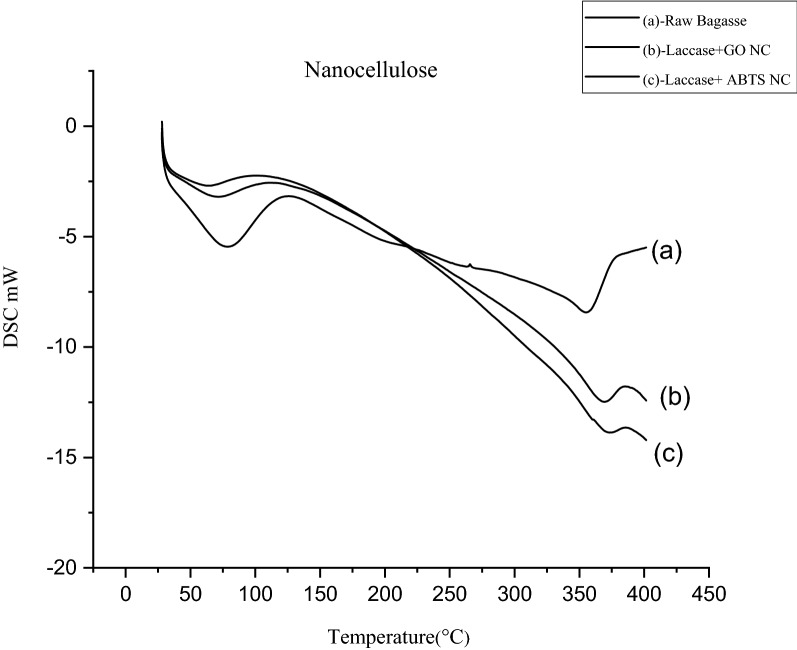
DSC thermograms for (**a**) raw bagasse, (**b**) laccase + GO nanocellulose and **(c)** laccase + ABTS nanocellulose

Figure 10 shows the DSC thermograms of raw bagasse and the nanocellulose product obtained from laccase + GO and laccase + ABTS. All the DSC thermograms exhibited two distinct endothermic changes within the range of temperature studied. The nature of endotherms, however, is quite characteristic of the composition of the material and differs from each other (Mandal and Chakrabarty 2011). The initial endotherm occurs in all the cases at a temperature much lower than 100 °C, and stands for the loss of moisture due to evaporation. For raw bagasse the initial degradation starts at 55 °C and for nanocellulose extracted from laccase + GO the initial decomposition starts at 71 °C, whereas for the nanocellulose extracted from laccase + ABTS the initial decomposition starts at 62 °C. Raw bagasse has various other constituents like lignin, hemicellulose, and other non-cellulosic components besides cellulose. All these hydrophilic substances help to retain moisture not only in greater proportion but also cause a variation in the sorptive forces holding this moisture. Therefore, the process of moisture loss for raw bagasse occurs over a wide range of temperatures compared to nanocellulose. The nanocellulose may be assumed to contain no hemicellulose, lignin content hence the nanocellulose may have absorbed moisture with uniform sorptive force and hence causing the moisture loss with a narrow range of temperature as observed from below Fig. 10. The second endotherm in each of the three cases is an indication of the course of fusion or melting which gives an idea of the nature of decomposition of the crystallites. The crystallinity increases from raw bagasse to nanocellulose. In the case of raw bagasse, we observe the fusion process occurring over a wide range of temperature from 311 to 359 °C. Raw bagasse contains various other constituents which have their characteristic melting range causing a wide temperature range for the raw bagasse material. In nanocellulose, a substantial proportion of the non-cellulosic materials are lost, and cellulose crystals gets re-arranged in a more compact crystal structure and hence the range narrows for laccase + GO (359.44–363.72 °C) and for laccase + ABTS (368.87–380.68 °C).

#### Thermo-gravimetric analysis (TGA)

**Fig. 11:**
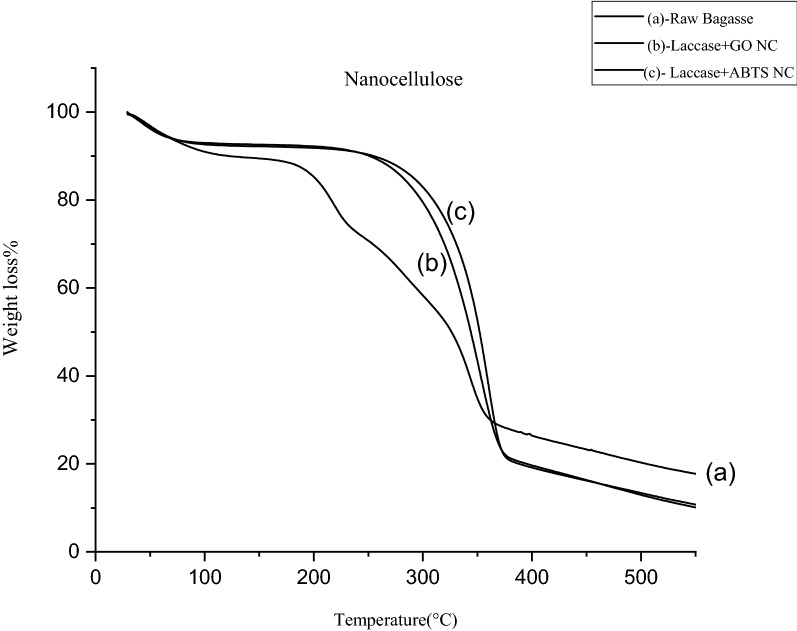
TGA comparison between **(a)** raw bagasse and **(b)** laccase + GO nanocellulose and **(c)** laccase + ABTS nanocellulose

The initial weight loss occurs for raw bagasse and nanocellulose at 30 °C. This is because of the loosely bound moisture on the surface of the materials. The chemisorbed water is found to be given off at approx. at 120 °C for the raw bagasse and nanocellulose. In Fig. 11, the degradation of the raw bagasse is observed to be at 226 °C and the rate of degradation reaches its peak at 335.65 °C as reported in our earlier work (Ramesh et al. 2021). The degradation of nanocellulose is associated with an additional hump, where there is a removal of all other components which increases the cellulose crystallinity as observed by XRD results and the crystals become denser and more compact (Mandal and Chakrabarty 2011). This re-orientation of crystals leads to the rise in the onset temperature of degradation for nanocellulose. For laccase + GO nanocellulose the onset temperature was observed at 319 °C with the maximum peak at 342.72 °C, whereas for laccase + ABTS nanocellulose the onset temperature was observed at 327.65 °C with the maximum peak at 345.07 °C. Generally, thermal degradation involves dehydration, depolymerization, and decomposition of glycosyl units and then the formation of a charred residue. The residual char formation was seen to be more in the case of nanocellulose probably due to the nano-dimensions and availability of free ends of the chains. Also, the higher crystalline nature (cellulose crystal) of the nanocellulose increases the proportion of carbon, therefore the formation of char residue increases as carbon content increases for the nanocellulose (Kumar et al. 2014).

## Conclusions

- Methods to improve the effect of greener routes using enzyme laccase, for producing nanocellulose with better properties were investigated. The role of mediator and additional enzymes was also investigated, respectively.
- Laccase was used in combination with glucose oxidase (GO) enzyme, which acts as an auxiliary enzyme and 2,2′-azinobis (3-ethylbenzthiazoline-6-sulfonate) (ABTS) which is a mediator molecule. A detailed comparison of laccase with GO and laccase with ABTS using analytical techniques such as gravimetric analysis, FT-IR, XRD, SEM, DSC, TGA and particle size analysis was performed.
- The addition of the moderators to the laccase enzyme significantly improved the performance of laccase. Maximum lignin removal and the highest cellulose content (62.1%) were found when ABTS (at 1.4 mM) was used with laccase enzyme. In terms of cellulose crystallinity, ABTS (at 1.4 mM) and GO (at 0.15 mg ml^−1^) yielded comparable results. The crystallinity of cellulose increased to approx. 70% for both cases. Thus, optimal values of ABTS (at 1.4 mM) and GO (at 0.15 mg ml^−1^) were chosen for nanocellulose production.
- A detailed comparison of nanocellulose obtained from laccase + ABTS and laccase + GO was performed. The cellulose content (82.22%) and cellulose crystallinity (61.71%) were found to be slightly better in the case of nanocellulose obtained from laccase + ABTS.
- However, it was found that the laccase + GO performed better as compared to that of ABTS when PSA and SEM analysis were performed. The fibres from GO treatment had an even and cleaner topology with negligent agglomeration which confirms the removal of all other constituents. Also, a narrow particle size distribution range in the particle size analysis was observed in nanocellulose obtained after laccase + GO enzyme treatment and the dimensions were found to be 339 nm (99% by vol.)
- From DSC thermograms, a more compact and denser cellulose crystal structure was seen for laccase + GO as a comparatively narrower endotherm range was noted.
- Based on the detailed analysis, nanocellulose obtained from laccase and glucose oxidase treatment demonstrated superior properties. The studies indicated that the efficacy of laccase was found to remarkably improve in presence of auxiliary enzyme glucose oxidase and nanocellulose with improved properties was obtained from sugarcane bagasse.

In the current study, laccase activity increased in presence of both moderators, ABTS and glucose oxidase. However, while ABTS improved the process by removing maximum lignin content, a superior quality of nanocellulose was obtained in the case of glucose oxidase. This demonstrates the differences in the mode of action of the laccase enzyme in presence of the two moderators. While ABTS seems to improve the laccase substrate specificity, glucose oxidase was additionally found to remove other fibres to yield nanocellulose with a smooth finish. Further studies to establish differences in mechanism of actions of the two moderators would be implemented to understand differences in lignin degradation.

## Data Availability

The datasets used and/or analysed during the current study are available from the corresponding author on reasonable request.

## Abbreviations

ABTS: 2,2′-Azinobis (3-ethylbenzthiazoline-6-sulfonate)
GO: Glucose oxidase
SEM: Scanning electron microscopy
FT-IR: Fourier-transform infrared
DSC: Differential scanning calorimeter
TGA: Thermo-gravimetric analysis
PSA: Particle size analyzer
XRD: X-ray diffraction

## References

[CR1] Abraham E, Deepa B, Pothen LA (2013). Environmental friendly method for the extraction of coir fibre and isolation of nanofibre. Carbohydr Polym.

[CR2] Agrawala M, Naik R, Shetgara S, Purnima D (2019). Surface treatment of jute fibre using eco-friendly method and its use in PP composites. Mater Today Proc.

[CR3] Azzam AM (1989). Pretreatment of cane bagasse with alkaline hydrogen peroxide for enzymatic hydrolysis of cellulose and ethanol fermentation. J Environ Sci Heal Part B.

[CR4] Chandra R, Yadav S, Kumar V (2015). Microbial degradation of lignocellulosic waste and its metabolic products. Environ Waste Manag.

[CR5] Chanoca A, de Vries L, Boerjan W (2019). Lignin engineering in forest trees. Front Plant Sci.

[CR6] Christopher LP, Yao B, Ji Y (2014). Lignin biodegradation with laccase-mediator systems. Front Energy Res.

[CR7] Curl F, Fiskari JPT, Mcdonough TJ et al (2001) Formation of quinonoid structures in laccase-mediator reactions. Inst Pap Sci Technol Atlanta, Georgia, pp 894

[CR8] De Campos A, Correa AC, Cannella D (2013). Obtaining nanofibers from curauá and sugarcane bagasse fibers using enzymatic hydrolysis followed by sonication. Cellulose.

[CR9] de Oliveira FB, Bras J, Pimenta MTB (2016). Production of cellulose nanocrystals from sugarcane bagasse fibers and pith. Ind Crops Prod.

[CR10] de Rocha GJM, Nascimento VM, Gonçalves AR (2015). Influence of mixed sugarcane bagasse samples evaluated by elemental and physical-chemical composition. Ind Crops Prod.

[CR11] Fan M, Dai D, Huang B (2012). Fourier transform—mater anal.

[CR12] Flórez-Pardo LM, Paz ASP, Galán JEL, Figueroa-Oviedo JI (2015). Using a mediator system to increase the delignification of sugarcane residues with fungal enzymes. CT&F Cienc Tecnol Futuro.

[CR13] Ghazy MB, Esmail FA, El-Zawawy WK (2016). Extraction and characterization of nanocellulose obtained from sugarcane bagasse as agro-waste. J Adv Chem.

[CR14] Gupta GK, Shukla P (2020). Lignocellulosic biomass for the synthesis of nanocellulose and its eco-friendly advanced applications. Front Chem.

[CR15] Karimi K, Taherzadeh MJ (2016). A critical review of analytical methods in pretreatment of lignocelluloses: composition, imaging, and crystallinity. Bioresour Technol.

[CR16] Karp SG (2015). Statistical optimization of laccase production and delignification of sugarcane bagasse by *Pleurotus ostreatus* in solid-state fermentation. Biomed Res Int.

[CR17] Khalid MY (2021). Recent advances in nanocellulose-based different biomaterials: types, properties, and emerging applications. J Mater Res Technol.

[CR18] Khalid MY, Al Rashid A, Arif ZU (2021). Natural fiber reinforced composites: sustainable materials for emerging applications. Results Eng.

[CR19] Kumar A, Negi YS, Bhardwaj NK, Choudhary V (2012). Synthesis and characterization of methylcellulose/PVA based porous composite. Carbohydr Polym.

[CR20] Kumar A, Negi YS, Choudhary V, Bhardwaj NK (2014). Characterization of cellulose nanocrystals produced by acid-hydrolysis from sugarcane bagasse as agro-waste. J Mater Phys Chem.

[CR21] Leonowicz A (2001). Fungal laccase: properties and activity on lignin. J Basic Microbiol.

[CR22] Luzzi SC, Artifon W, Piovesan B (2017). Pretreatment of lignocellulosic biomass using ultrasound aiming at obtaining fermentable sugar. Biocatal Biotransform.

[CR23] Machado E (2020). Reduction in lignin content and increase in the antioxidant capacity of corn and sugarcane silages treated with an enzymatic complex produced by white rot fungus. PLoS ONE.

[CR24] Mandal A, Chakrabarty D (2011). Isolation of nanocellulose from waste sugarcane bagasse (SCB) and its characterization. Carbohydr Polym.

[CR25] Melati RB et al (2017) Sugarcane bagasse: production, composition, properties, and feedstock potential. Sugarcane prod syst uses econ importance. pp 1–38. https://repositorio.unesp.br/handle/11449/174692

[CR26] Michelin M, Gomes DG, Romaní A (2020). Nanocellulose production: exploring the enzymatic route and residues of pulp and paper industry. Molecules.

[CR27] Moilanen U, Kellock M, Várnai A (2014). Mechanisms of laccase-mediator treatments improving the enzymatic hydrolysis of pre-treated spruce. Biotechnol Biofuels.

[CR28] Pamidipati S, Ahmed A (2017). Degradation of lignin in agricultural residues by locally isolated fungus *Neurospora discreta*. Appl Biochem Biotechnol.

[CR29] Pamidipati S, Ahmed A (2019). Cellulase stimulation during biodegradation of lignocellulosic residues at increased biomass loading. Biocatal Biotransform.

[CR30] Pereira PHF, Voorwald HCJ, Cioffi MOH (2011). Sugarcane bagasse pulping and bleaching: thermal and chemical characterization. BioResources.

[CR31] Pereira Ramos L (2003). The chemistry involved in the steam treatment of lignocellulosic materials. Quim Nova.

[CR32] Rahikainen J, Mikander S, Marjamaa K (2011). Inhibition of enzymatic hydrolysis by residual lignins from softwood-study of enzyme binding and inactivation on lignin-rich surface. Biotechnol Bioeng.

[CR33] Rahimi Kord Sofla M, Brown RJ, Tsuzuki T, Rainey TJ (2016). A comparison of cellulose nanocrystals and cellulose nanofibres extracted from bagasse using acid and ball milling methods. Adv Nat Sci Nanosci Nanotechnol.

[CR34] Rajak RC, Banerjee R (2015). Enzymatic delignification: an attempt for lignin degradation from lignocellulosic feedstock. RSC Adv.

[CR35] Ramesh S, Doddipatla P, Pamidipati S (2021). Optimization of parameters for biological pre-treatment route for the production of nanocellulose from sugarcane bagasse. Biomass Convers Biorefinery.

[CR36] Rencoret J, Pereira A, del Río JC, Martínez AT, Gutiérrez A (2017). Delignification and saccharification enhancement of sugarcane byproducts by a laccase-based pretreatment. ACS Sustain Chem Eng.

[CR37] Rodríguez-Escribano D, de Salas F, Pardo I, Camarero S (2017). High-throughput screening assay for laccase engineering toward lignosulfonate valorization. Int J Mol Sci.

[CR38] Siqueira G, Tapin-Lingua S, Bras J (2010). Morphological investigation of nanoparticles obtained from combined mechanical shearing, and enzymatic and acid hydrolysis of sisal fibers. Cellulose.

[CR39] Szklarz G, Leonowicz A (1986). Cooperation between fungal laccase and glucose oxidase in the degradation of lignin derivatives. Phytochemistry.

[CR40] Tadesse MA, D’Annibale A, Galli C (2008). An assessment of the relative contributions of redox and steric issues to laccase specificity towards putative substrates. Org Biomol Chem.

[CR41] Tavares AP, Cristovao RO, Loureiro JM (2008). Optimisation of reactive textile dyes degradation by laccase–mediator system. J Chem Technol Biotechnol.

[CR42] Tsegaye B, Balomajumder C, Roy P (2018). Biodelignification and hydrolysis of rice straw by novel bacteria isolated from wood feeding termite. 3 Biotech.

[CR43] Viera RGP, Filho GR, de Assunção RMN (2007). Synthesis and characterization of methylcellulose from sugar cane bagasse cellulose. Carbohydr Polym.

[CR44] Witayakran S, Ragauskas AJ (2009). Synthetic applications of laccase in green chemistry. Adv Synth Catal.

[CR45] Wulandari WT, Rochliadi A, Arcana IM (2016). Nanocellulose prepared by acid hydrolysis of isolated cellulose from sugarcane bagasse. IOP Conf Ser Mater Sci Eng.

[CR46] Yarbrough JM, Zhang R, Mittal A (2017). Multifunctional cellulolytic enzymes outperform processive fungal cellulases for coproduction of nanocellulose and biofuels. ACS Nano.

[CR47] Yu S, Sun J, Shi Y (2021). Nanocellulose from various biomass wastes: its preparation and potential usages towards the high value-added products. Environ Sci Ecotechnol.

